# Phenolic Compounds and Biological Activity of Selected *Mentha* Species

**DOI:** 10.3390/plants10030550

**Published:** 2021-03-15

**Authors:** Sanja Ćavar Zeljković, Jana Šišková, Karolína Komzáková, Nuria De Diego, Katarína Kaffková, Petr Tarkowski

**Affiliations:** 1 Centre of the Region Haná for Biotechnological and Agricultural Research, Department of Genetic Resources for Vegetables, Medicinal and Special Plants, Crop Research Institute, Šlechtitelů 29, 78371 Olomouc, Czech Republic; kaffkova@genobanka.cz (K.K.); or petr.tarkowski@upol.cz (P.T.); 2 Centre of the Region Hana for Biotechnological and Agricultural Research, Palacky University Olomouc, Šlechtitelů 27, 78371 Olomouc, Czech Republic; jana.siskova44@seznam.cz (J.Š.); komzakova.kaja@seznam.cz (K.K.); nuria.de@upol.cz (N.D.D.)

**Keywords:** *Mentha* sp., phenolic compounds, LC–MS/MS, antioxidant activity, tyrosinase inhibition activity

## Abstract

*Mentha* species are widely used as food, medicine, spices, and flavoring agents. Thus, chemical composition is an important parameter for assessing the quality of mints. In general, the contents of menthol, menthone, eucalyptol, and limonene comprise one of the major parameters for assessing the quality of commercially important mints. Building further on the phytochemical characterization of the quality of *Mentha* species, this work was focused on the composition of phenolic compounds in methanolic extracts. Thirteen *Mentha* species were grown under the same environmental conditions, and their methanolic extracts were subjected to the LC–MS/MS (liquid chromatography–tandem mass spectrometry) profiling of phenolics and the testing their biological activities, i.e., antioxidant and tyrosinase inhibition activities, which are important features for the cosmetic industry. The total phenolic content (TPC) ranged from 14.81 ± 1.09 mg GAE (gallic acid equivalents)/g for *Mentha cervina* to 58.93. ± 8.39 mg GAE/g for *Mentha suaveolens*. The antioxidant activity of examined *Mentha* related with the content of the phenolic compounds and ranged from 22.79 ± 1.85 to 106.04 ± 3.26 mg TE (Trolox equivalents)/g for *M. cervina* and *Mentha x villosa*, respectively. Additionally, *Mentha pulegium* (123.89 ± 5.64 mg KAE (kojic acid equivalents)/g) and *Mentha x piperita* (102.82 ± 15.16 mg KAE/g) showed a strong inhibition of the enzyme tyrosinase, which is related to skin hyperpigmentation. The most abundant compound in all samples was rosmarinic acid, ranging from 1363.38 ± 8323 to 2557.08 ± 64.21 μg/g. In general, the levels of phenolic acids in all examined mint extracts did not significantly differ. On the contrary, the levels of flavonoids varied within the species, especially in the case of hesperidin (from 0.73 ± 0.02 to 109. 39 ± 2.01 μg/g), luteolin (from 1.84 ± 0.11 to 31.03 ± 0.16 μg/g), and kaempferol (from 1.30 ± 0.17 to 33.68 ± 0.81 μg/g). Overall results indicated that all examined mints possess significant amounts of phenolic compounds that are responsible for antioxidant activity and, to some extent, for tyrosinase inhibition activity. Phenolics also proved to be adequate compounds, together with terpenoids, for the characterization of *Mentha* sp. Additionally, citrus-scented *Mentha* x *villosa* could be selected as a good candidate for the food and pharmaceutical industry, especially due its chemical composition and easy cultivation, even in winter continental conditions.

## 1. Introduction

Phenolic compounds belong to the most frequent and widespread groups of plant metabolites, with more than 8000 identified structures to date. The importance of plant phenolics (flavonoids, phenolic acids, etc.) is widely known in human health care. These bioactive plant secondary metabolites have been an inexhaustible source of scientific research, including their isolation from plant extracts, the assessment of their chemical structure, and the characterization of a wide array of biological properties [1,2]. Since ancient times, the human race has been exploring the potential of plants to improve their health, often attributed to the presence of phenolic compounds with strong antioxidant properties. The antioxidant ability of phenolic components occurs mainly through a redox mechanism and allows the components to act as reducing agents, hydrogen donors, singlet oxygen quenchers, and metal chelators [3]. This antioxidant mechanism has an important role in the reduction of lipid oxidation in tissues—both plant and animal. Therefore, plant phenolics are beneficial because they not only conserve the quality of plant-based food and food products but also reduce the risk of the development of some human disorders, such as cardiovascular diseases, arteriosclerosis, cancer, diabetes, cataract, disorders of cognitive function, and neurological diseases [1,2,3].

Several *Mentha* species are official drugs described in European pharmacopeia [4] and important raw materials for the pharmaceutical, cosmetic, perfume, and food industries [5,6,7,8]. The majority of them are cultivated in India and the USA, but some are cultivated in China and Europe. Mint is mainly cultivated for its essential oils (which are used as flavoring agents, beverage bases, and bee pasture plants) but can be interesting as ornamental plant [7,9]. Demand for mint and its products continues to steadily rise, driven by the worldwide growth of a broad category of fast-moving consumer goods in which mint is used in cosmetics for the production of toothpaste, mouthwashes, shampoos, shaving creams, etc. Both extracts and essential oils show a broad spectrum of activities that are beneficial to humans. Due to their natural origin and bioactivities, mint-derived products could become a great alternative to artificial preservatives and could find a wide range of applications for the shelf-life extension of foods [9,10]. Many of the biological and pharmacological effects of the *Mentha* plants are related to volatile monoterpenes *p*-menthane and carvone, as well as their derivatives. These oxygenated monoterpenoids are responsible for the specific aroma and taste of different mint species [10]. However, mint tea infusions have been used since ancient times to treat different disorders such as gastrointestinal tract discomfort, migraines, and diseases of the upper respiratory tract [11]. Moreover, there are dozens of commercial bile ducts, gastrointestinal and liver remedies, hypnotics, sedatives, and laxatives, which include extracts of different *Mentha* species [9]. The major constituents of herbal infusions and alcoholic extracts are non-volatile, polar phenylpropanoids, mainly phenolic acids such as rosmarinic (RA) and chlorogenic (ChA) acid, but also in flavonoids such as apigenin (API) and luteolin (LUT) [11,12].

**Figure 1:**
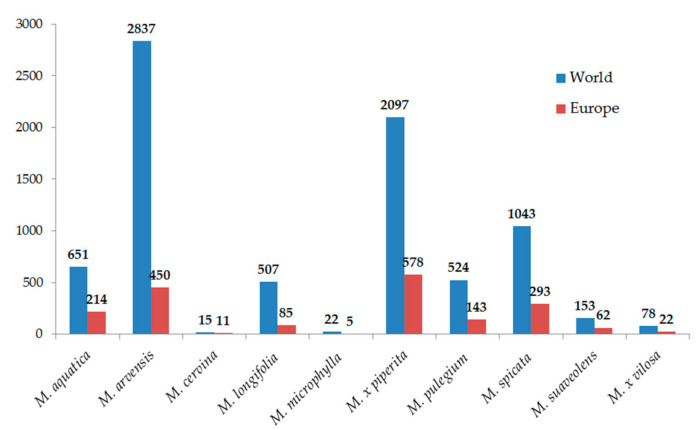
The number of studies on *Mentha* species in the last twenty years (Scopus, accessed on 28 December 2020).

According to the number of published articles, it might be concluded that *Mentha* species have been thoroughly investigated from their morphology and chemistry to a wide array of biological activities, and uses in different aspects of the food and pharmaceutical industries. Still, this genus is in the focus of scientists all over the world. Figure 1 shows a literature survey from the last twenty years on the *Mentha* species used in this study, i.e., the numbers of papers published referring to each particular species. Interestingly, the highest number of papers published for each mint, except for *Mentha microphylla*, was in 2020, which clearly shows that plants of this genus are being spotlighted among other medicinal and aromatic plants. Though the Americas and Asia are the biggest producers and consumers of mints, approximately one-third of studies have been European (Figure 1), with Italy and Spain as the leading countries.

Species of the genus *Mentha* are widely distributed in temperate and sub-temperate regions. The understanding of the systematics of this genus has been an extremely challenging and very complex task. Many studies have elucidated these problems [5,13,14,15,16]. Taxonomic difficulties are mainly caused by a high incidence of polyploidy, variations in base chromosome number, diverse morphologies, vegetative propagation, and frequent interspecific hybridization [17,18]. Therefore, chemical analysis is an important tool to help discriminate between the species of this genus [14,19], although most of the commercially important mints are hybrids or amphiploids. The genus *Mentha* (Lamiaceae) consists of eighteen species and eleven hybrids, which are separated into four sections: *Pulegium*, *Tubulosae*, *Eriodentes,* and *Mentha* [5]. In general, species of the genus *Mentha* are described as aromatic perennial plants with rhizomes or rhizomatous stems. Individual species differ in plant height (from small *Mentha x piperita* var. *citrata* to high *Mentha rotundifolia* and *Mentha longifolia* in leaf color (from light green, such as *Mentha spicata*, to dark green with hints of purple, such as *M. x piperita*), leaf length (short like *Mentha arvensis* to long like *M. longifolia*), leaf surface (from glabrous like *M. spicata* to hairy like *M. longifolia*), the shape of inflorescence (from the remote verticillasters of *Mentha pulegium* to the dense spike-like inflorescence of *Mentha aquatica*), and flower color (from nearly white to purple) [20,21].

The majority of *Mentha* species tolerate a wide range of soil chemistry and conditions. The most important factors are neutral pH, organic content, overall water-holding capacity, and drain ability [7]. However, environmental conditions not only strongly affect the quality of *Mentha* essential oil but also phenylpropanoid composition. For example, the essential oils of *M. arvensis* from Brazil, Taiwan, and China significantly differ in the contents of the major compounds menthol, and menthone. Additionally, considerable differences in the oil from *M. piperita* of European origin were also found [22]. The negative effect of environmental stress on mint essential oil quality has been well-studied [1]. In general, long warm days promote both phenolics and monoterpene synthesis and accumulation. Additionally, plant harvesting is an important factor that influences essential oil quality and quantity [23]. Plants harvested at the flowering stage usually have a higher concentration of menthol and its derivatives than plants harvested in the bud formation stage.

The quality control of plant foods has increased in many fields of food science and technology because the introduction of modern chromatographic techniques, like gas and liquid chromatography coupled with mass spectrometry detectors, can provide the detailed chemical composition of complex matrixes in a very short time [24]. Therefore, this study aimed to assess and compare the detailed phenolic composition of commercially important mint species of European origin that were grown under the same environmental conditions (continental climate with warm, dry summers and fairly cold winters). Additionally, profiles of volatile compounds were acquired and compared with literature data. Finally, the antioxidant and tyrosinase inhibition activities of isolated essential oils and methanolic extracts were assayed.

## 2. Results and Discussion

### 2.1. Phytochemical Analysis of Selected Mentha Species

The total phenolic content (TPC) and total flavonoid content (TFC) of the methanolic extracts of examined mints were firstly assayed spectrophotometrically. These preliminary results presented general differences in phenolic contents between investigated species. Values of gallic acid equivalents (GAEs) for the total phenolic content and quercetin equivalents (QEs) for flavonoid content are summarized in Supplementary Table S1. The lowest values for both total phenolics and flavonoids were found in the extract of *Mentha cervina* (14.81 ± 1.09 mg GAE/g and 3.65 ± 0.037 mg QE/g), while the extract of *Mentha suaveolens* was found to be richest in total phenolics (58.93 ± 8.39 mg GAE/g) and *M. longifolia* was found to be richest in flavonoids (16.83 ± 1.45 mg QE/g).

Additionally, the Czech genotype of *Mentha x villosa* showed significant amounts of phenolic compounds (Supplementary Table S1). This can be attributed to the fact that this genotype is related to *M. suaveolens*, which originated by crossing this genotype with *M. spicata*. A literature overview showed that *M. piperita* and *M. spicata* are also quite rich in phenolics (Table 1), which corresponded with the results presented here (Table S1 in Supplementary Material). Among *M. x piperita* genotypes, the *M. x piperita var. citrata* contained the lowest amount of phenolics, probably because this species is genetically very close to *M. aquatica* [22]. In general, values in the present study corresponded with those found in the literature, although differences could be attributed to environmental, phenological, and genetic factors. For example, plants of Mediterranean origin, in general, possess higher amounts of phenolics in comparison to the same species grown under continental conditions (Table 1).

In general, solid conclusions on the available literature data face obstacles caused by methodology problems, such as missing information on the definition of the analyzed plant organ and the phenological stage of the plant. Nevertheless, previous studies confirmed that *M. cervina* possesses the lowest values in total phenolic content, while *M. aquatica* possesses the highest. The biggest variations were found for *M. x piperita*, which was expected because there are more different cultivars of this species included in Table 1. Moreover, this study showed that the alcoholic extract of *M. suaveolens* possesses the highest amounts of phenolic compounds, while, according to the available literature, *M. suaveolens* from Slovakia [56] is not as rich as the one investigated here. Therefore, a comparison of the chemical content of biologically active compounds in different species of the same genus, such as phenolics, should be performed for plants that are grown under the same environmental conditions and harvested at the same phenological stage.

**Figure 2:**
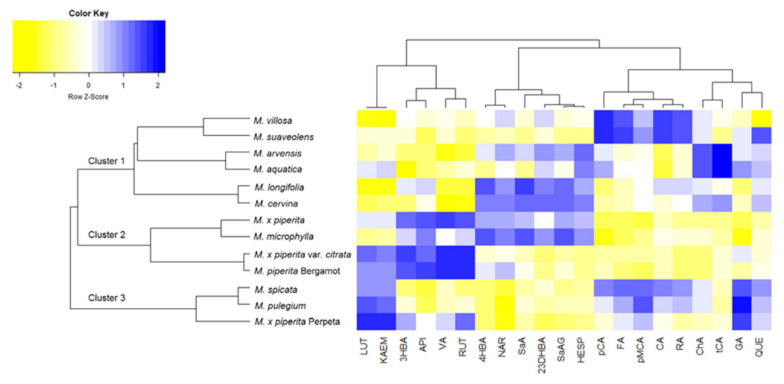
Heatmap of the levels of phenolic compounds in different *Mentha* species. LUT: luteolin; KAEM: kaempferol; 3HBA: 3-hydroxybenzoic acid; API: apigenin; VA: vanillic acid; RUT: rutin; 4HBA: 4-hydroxybenzoic acid; NAR: naringenin; SaA: salicylic acid; 23DHBA: 2,3-dihydroxybenzoic acid; SaAG: salicylic acid 2-O-β-glucoside; HESP: hesperidin; pCA: *p*-coumaric acid; FA: ferulic acid; pMCA: *p*-methyl coumarate; CA: caffeic acid; RA: rosmarinic acid; ChA: chlorogenic acid; tCA: trans-cinnamic acid; GA: gallic acid; QUE: quercetin.

Furthermore, to identify and quantify particular phenolic constituents, each methanolic extract was subjected to detailed LC–MS/MS analysis. The separation of the analytes was done on a reversed-phase C18 column, and their quantification was performed in MRM (multiple reaction monitoring) modes via the isotopic dilution method. The identity of all analytes was confirmed by two qualifying MRM transitions and retention times. Results are summarized in Supplementary Table S2. Among the 33 investigated analytes, *Mentha* methanolic extracts contained seven hydroxybenzoic acids, six hydroxycinnamic acids, and seven flavonoids. For better visualization and to compare the thirteen selected *Mentha* species, a heatmap clustering the compounds and species according to their correlation was constructed (Figure 2). For that, the levels of phenolics (Supplementary Table S2) were transformed using common logarithm (log10). After that, data were evaluated for the number of clusters using the silhouette method in RStudio. As results, the thirteen *Mentha* sp. were divided into three main clusters (Figure 2). The first one was also subclustered into three groups, while the other two were subclustered into two groups. In the first, there were *M. villosa* and *M. suaveolens,* which presented the highest levels of RA, caffeic acid (CA), *p*-methyl coumarate (pMCA), ferulic acid (FA), and *p*-coumaric acid (pCA) (Figure 2). RA was the main compound found in all examined extracts, with levels ranging from 1363.38 ± 83.23 μg/g (*M. cervina*) to 2557.08 ± 64.21 μg/g (*M. suaveolens*) (Supplementary Table S2). The other two sub-clusters were composed of *M. arvensis*, *M. aquatica*, *M. longifolia*, and *M. cervina,* mainly because of their high levels of ChA, *trans*-cinnamic acid (tCA), and quercetin (QUE), and lower values of vanillic acid (VA) and rutin (RUT). Opposite results were observed in all *M. piperita* sp. and *M. microphylla*, located in the second cluster (Figure 2). They were also separated into two subclusters; first with *M. piperita* and *M. microphylla* and second with *M. x piperita* var. *citrata* and *M. x piperita* Bergamot, mainly due to the high levels of 4-hydroxybenzoic acid (4HBA), salicylic acid (SaA), salicylic acid-2-*O*-β-glucoside (SaAG), and the flavonoids naringenin (NAR) and hesperidin (HESP) in the first ones. Among hydroxybenzoic acids, SaAG was the most abundant representative, ranging from 3.27 ± 0.16 μg/g (*M. spicata*) to 149.79 ± 8.14 μg/g (*M. arvensis*) (Supplementary Table S2). Additionally, HESP (up to 121.95 ± 2.35 μg/g) and LUT (up to 31.03 ± 0.16 μg/g) were the most abundant flavonoids found in all examined extracts. To the best of our knowledge, this study presents the most comprehensive analysis of phenolic compounds of these *Mentha* species.

In general, the results presented here were in agreement with those found in the literature (Table 1). RA is the main phenolic acid found in this genus [1,12,32,54], followed by the flavone LUT [1,12,27,57] and the flavanone eriocitrin [1,34,46]. The levels of phenolic compounds, as well as volatile terpenoids, are dependent on the phenological stage of the plant. According to Fletcher et al. [58], flowering plays a crucial role in the reduction of rosmarinic acid levels in spearmint (*M. spicata*) and peppermint (*M. piperita*). Though studied *Mentha* species were collected at the flowering stage, they still possessed significant amounts of rosmarinic acid. Many the traditional and uses in official medicine of peppermint, spearmint, and other mint species are closely related to the presence of these phenolic compounds. Infusions obtained from leaves of several *Mentha* species are frequently used as folk remedies for the treatment of anorexia, hypertension, ulcerative colitis, etc. [1,5,7,9]. In the plant, RA serves as a defense compound against pathogens and herbivores, while its most prominent benefits for humans are antibacterial, anti-inflammatory, and antiviral activities [59].

**Figure 3:**
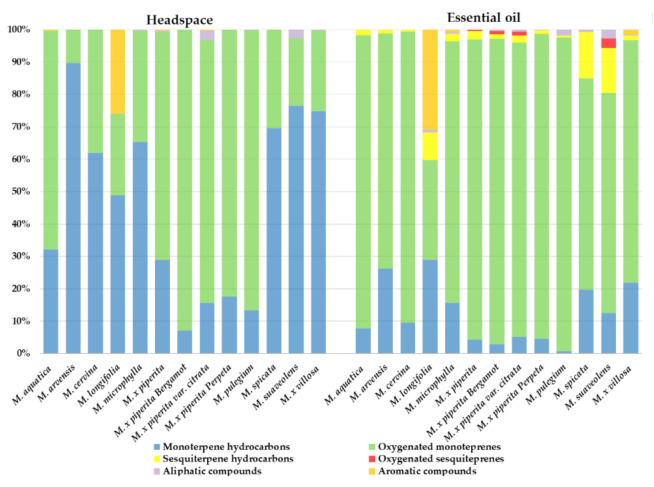
Volatile profiles of selected *Mentha* species.

Since the quality of these aromatic species is mainly characterized by the composition of their essential oils, dried plant material was subjected to static headspace analysis and hydro-distillation for the isolation of the essential oils. The percentage contents of both headspace and essential oil analyzed via GC–MS are summarized in Figure 3, while the detailed compositions of *Mentha* volatiles are presented in Supplementary Table S3. The major classes of headspaces and essential oils in all investigated species were monoterpene hydrocarbons, such as limonene (LIM), γ-terpinene (TERP), and oxygenated monoterpenes (i.e., 1,8-cineole (CIN), linalool acetate (LINAC), pulegone (PUL), piperitenone (PIP), and piperitenone oxide (PIPOX)), and significant amounts of the volatile phenol thymol (THY) were found in the oil of *M. longifolia* (Figure 3).

**Figure 4:**
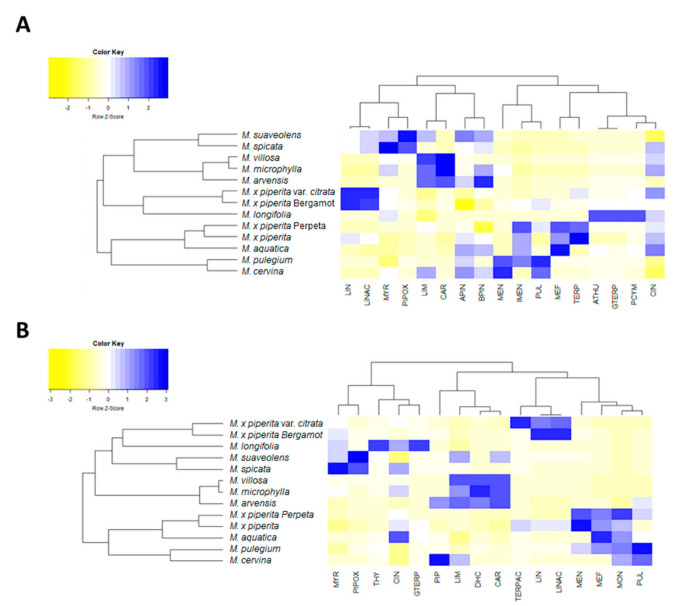
Heat maps of the headspaces (**A**) and essential oils (**B**) in thirteen different *Mentha* species. LIN: linalool; LINAC: linalyl acetate; MYR: myrcene; PIPOX: piperitenone oxide; LIM: limonene; CAR: carvone; APIN: *α*-pinene; BPIN: *β*-pinene; MEN: menthone; IMEN: isomenthone; TERP: terpinen-4-ol; ATHU: *α*-thujene; GTERP: *γ*-terpinene; PCYM: *p*-cymene; CIN: 1,8-cineole; THY: thymol; PIP: piperitenone; DHC: dihydrocarvone; TERPAC: *α*-terpinyl acetate; MEH: menthofuran; MON: menthone; PUL: pulegone.

To go further in the evaluation regarding how essential oil composition could be used as a chemotype marker in *Mentha*, two more heatmaps were prepared. As before, the levels of the essential oil components and terpenoids were transformed using the log10 and the number of clusters using the silhouette method in RStudio. Only compounds whose levels were >5% of the oil were used in this analysis. However, for these type of compounds, there was not a clear clustering of the *Mentha* sp., mainly because both the compounds and their levels were different for most of the thirteen evaluated species (Figure 4). For example, it can be seen that the *M. piperita* sp. in this case were not clustered all together but in very distant ones when the headspace samples were analyzed (Figure 4A). Similar results were obtained for the essential oils (Figure 4B). However, the rest of the clusters were similar between both heat-maps. For example, *M. villosa*, *M. microphylla,* and *M. arvensis* were sub-clustered together, and *M. suaveolens* and *M. spicata* formed another sub-cluster close to them (Figure 3). The fact that *M. microphylla* was clustered with different *Mentha* species when the terpenoids or phenols were analyzed pointed to a combination of both as the best strategy to construct the chemotype in mint plants.

Considering the essential oil composition, plants of the genus *Mentha* show great chemical polymorphism, both intra- and inter-species. Many factors interfere with essential oil composition, including environmental, phenological, the plant part used for essential oil extraction, and the freshness of the material. [7,9]. This fact makes the need to using complementary compounds such as phenolics for a better characterization of *Mentha* sp. more important. Additionally, in the presented work, some of the investigated species showed the same or similar chemical composition of essential oils when compared with those found in the literature, namely *M. aquatica* [7,60,61], *M. cervina* [7,62], *M. suaveolens* [7,63], *M. x piperita* [7,16,60], *M. pulegium* [7,16,60], and *M. x villosa* [60]. The major volatiles of *M. spicata* are mainly carvone (CAR) and PUL [7,9] but the oil of the species studied here was of the piperitenone oxide (PIPOX) chemotype. This chemotype was also found in Greece [7]. Among the thirteen species investigated in this study, three new chemotypes were not described in the literature previously. The oil of the *M. arvensis* studied here was of the carvone/limonene chemotype, while the same species of European origin is usually of the piperitone/myrcene chemotype [16]. On the contrary, menthol is the main compound of the oil obtained from South American *M. arvensis* [7]. *Mentha longifolia* was characterized as 1,8-cineole/γ-terpinene chemotype (CIN/TERP) with significant amounts of aromatic phenol THY (Supplementary Table S3). The oil of *M. longifolia* shows a high variability [7,16,60], so discovering new chemotypes of this species is not unusual. Additionally, the *M. microphylla* studied here was of the CAR chemotype (Supplementary Table S32). On the contrary, to the best of our knowledge, there are just two records of the essential oil composition of *M. microphylla*, from Greece [64] and Italy [65], and both of them were of the PIPOX chemotype.

In general, *Mentha* essential oils with a high content of LIM, which has a pleasant lemon scent and is used in a wide range of cosmetic and food products or additives to industrial solvents, are of commercial interest [5,6,7]. Five out of thirteen investigated mints contained significant amounts of this monoterpene, i.e., *M. cervina, M. x villosa*, *M. arvensis*, *M. microphylla*, and *M. suaveolens* (Supplementary Table S2). Additionally, essential oils of *M. longifolia*, *M. x piperita*, and *M. aquatica* contained a high percentage of CIN, an oxygenated monoterpene that is commonly used in pharmaceutical preparations for the treatment of inflammation and respiratory system disorders [5,6,7].

### 2.2. Biological Activities of Selected Mentha Species

To characterize the pharmacological potential of selected mints grown under the same environmental conditions, methanolic extracts and essential oils were subjected to the testing of their abilities to reduce stable radicals and to inhibit the activity of the enzyme tyrosinase, which is related to skin hyperpigmentation disorders, such as melanoma and age spots.

Methanolic extracts and essential oils of all *Mentha* species were able to reduce stable DPPH radical into its neutral form. The results, shown as Trolox equivalents per g of the dry extract, are summarized in Table 2, and they ranged from 22.79 ± 1.85 mg TE/g (*M. cervina*) to 106.04 ± 3.26 mg TE/g (*M. x villosa*). It is well known that phenolic compounds from mints (but also from other plants) are the main carriers of antioxidant activities in alcoholic and aqueous plant extracts [1,12,25,32,41,66]. As expected, the presented data supported this fact. The Pearson correlation coefficient between total phenolic content and antioxidant activity of the methanolic extracts was 0.8307, while between total flavonoid content and antioxidant activity, it was slightly lower, i.e., 0.7105. The highest content of total phenolics was determined in the samples of *M. suaveolens*, *M. x villosa*, and *M. x piperita* Bergamot (Supplementary Table S1), and these species possessed the highest antioxidant activities.

**Figure 5:**
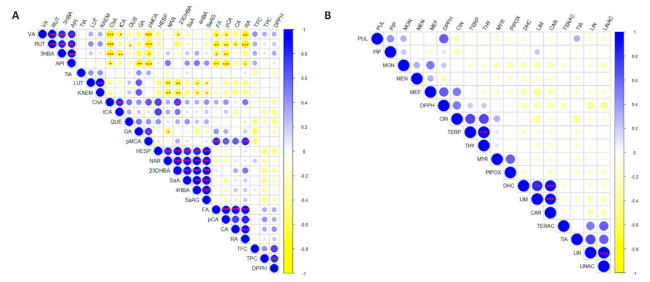
(**A**) Correlation matrix between phenolics, total flavonoids (TFC), total phenolics (TPC), and antioxidant (DPPH) and tyrosinase (TIA) activity in *Mentha* species. (**B**) Correlation matrix between terpenoids and DPPH and TIA in *Mentha* species. Red asterisks indicate the significance of the correlation (* *p* < 0.05; ** *p* < 0.01; *** *p* < 0.001). VA: vanillic acid; RUT: rutin; 3HBA: 3-hydroxybenzoic acid; API: apigenin; TIA: tyrosinase inhibition activity; LUT: luteolin; KAEM: kaempferol; ChA: chlorogenic acid; tCA: trans-cinnamic acid; QUE: quercetin; GA: gallic acid; pMCA: *p*-methyl coumarate; HESP: hesperidin; NAR: naringenin; 23DHBA: 2,3-dihydroxzbenyoic acid; SaA: salicylic acid; 4HBA: 4-hydroxybenzoic acid; SaAG - salicylic acid 2-*O*-β-D-glucoside; FA: ferulic acid; pCA: *p*-coumaric acid; CA: caffeic acid; RA: rosmarinic acid; TFC: total flavonoid content; TPC: total phenolic content; DPPH: antioxidant activity; PUL: pulegone; PIP: piperitenone; MON: menthone; MEN: menthol; MEF: menthofuran; CIN: 1,8-cineole; TERP: terpinen-4-ol; THY: thymol; MYR: myrcene; PIPOX: piperitenone oxide; DHC: dihydrocarveol; LIM: limonene; CAR: carvone; TERAC: α-terpinyl acetate; LIN: linalool; LINAC: linalyl acetate.

Finally, the correlation and its significance between the different groups of compounds and the antioxidant (DPPH) and tyrosinase inhibitory activity (TIA) was also analyzed and represented in two correlation matrices (Figure 5). First of all, there was a significant positive correlation between the TFC and TPC with the antioxidant activity (DPPH) (Figure 4A).

Additionally, essential oils possess notable antioxidant activities, although only the oil of *M. longifolia* contains strong antioxidant thymol (Supplementary Table S2). The antioxidant activity of the oils was in a wider range than their corresponding methanolic extracts (Table 2), which was in agreement with the fact that essential oils of the investigated *Mentha* species were much more diverse in their composition than their corresponding methanolic extracts. The highest antioxidant activity was recorded in the essential oil of *M. aquatica* (43.86 ± 0.33 mg TE/mL) and *M. pulegium* (40.01 ± 0.86 mg TE/mL), while *M. piperita var. citrata* had the lowest activity (2.71 ± 0.34 mg TE/mL). This could be explained by the fact that these oils contain significant amounts of strong radical scavengers, pulegone, 1,8-cineole, and menthone [1,5,7,22,67]. In fact, Pearson correlation coefficients for the antioxidant activities of the essential oil constituents were significant for menthofuran (MEF) and PUL (Figure 4B).

However, the values obtained from antioxidant assays could be used just for comparison between species. As can be seen in Table 1, the antioxidant activities of the alcoholic extracts of investigated *Mentha* species had been already studied elsewhere, but the values differed significantly. This can be explained by the fact that different methodologies and ways of presenting the data were used.

The investigated *Mentha* species also revealed some activity to inhibit the enzyme tyrosinase. Among thirteen species, the methanolic extracts of *M. pulegium* (123.89 ± 5.64 μg KAE/g) and *M. x piperita* (102.82 ± 15.16 μg KAE/g) showed the highest activities (Table 2). Though it has been suggested that the activity of the plant extracts against tyrosinase could be attributed to the presence of hydroxycinnamic acids and flavonoids [55], there was no positive correlation between the activity of the extracts and phenolic and flavonoid contents in the present study. However, other authors have observed no tyrosinase inhibition or very weak inhibition in mint extracts [50,68].

On the contrary, the essential oils of the investigated mints possessed some activity against this enzyme. Very high values of kojic acid equivalents revealed oils of *M. x piperita* Bergamot and *M. pulegium*. On the contrary, the oils of *M. spicata*, *M. aquatica*, and *M. longifolia* revealed very weak activity (Table 2). In general, it seems that terpenoids are stronger inhibitors of tyrosinase than phenolics. There are very strong positive correlations with the contents of the linalool (LIN), LINAC, and TIA of the oils. However, essential oils are very complex mixtures, and it is difficult to define whether their activity is based on the complex composition and potentially synergistic effect(s) of individual compounds present in each sample. Therefore, it has been suggested that the inhibitory activity of such complex mixtures cannot be easily explained by a few major compounds [69,70].

## 3. Materials and Methods

### 3.1. Plant Material

All *Mentha* plants used in this study were grown in the experimental field of Crop Research Institute, Department of Genetic Resources for Vegetables, Medicinal and Special Plants, Olomouc, Czechia (Olomouc-Holice: 9°37′ N, 17°17′ E, 209 m a.s.l. altitude).

**Figure 6:**
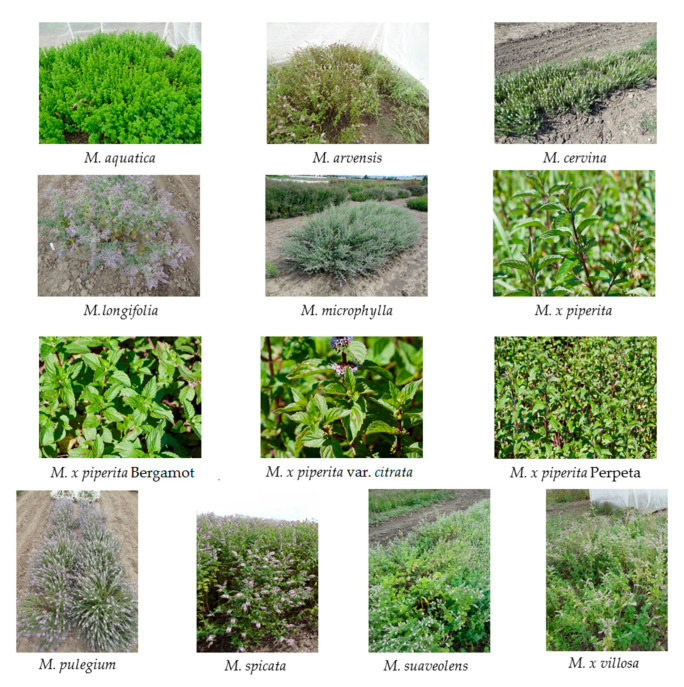
*Mentha* sp. used in the study.

It was already pointed in the Introduction section that species of the genus *Mentha* are very morphologically diverse. They vary in their height, leaf size, shape, and flower color. A brief overview of selected characteristics, such as the plant height, leaf description, and corolla colors of evaluated accessions, are summarized in Table 3. Additionally, photos of the plants taken in July–September 2018 are shown in Figure 6.

The lowest average recorded temperature was in May 2018 with 12.6 °C, and the highest average temperature recorded was in August 2018 with 17.9 °C. Maximum precipitation was recorded in July 2018 with 68 mm, and the minimum was in September 2018 with 25 mm (Czech Hydrometeorological Institute, Praha-Komořany, Czech Republic, 2020). The chemical analysis of the soil showed a nitrate content of 8.43 mg/kg, a potassium content of 648 mg/kg, a phosphorus content of 96 mg/kg, a calcium content of 5923 mg/kg, a magnesium content of 183 mg/kg, and an ammonia content of 6.15 mg/kg, with a pH of 6, and it was done by Agrolab, s.r.o., Troubsko, Czechia. The plants were harvested during the flowering stage (July–September 2018) and dried in the dark under air-flow, and (after the separation of stems) leaves were crushed and homogenized to a fine powder.

### 3.2. Isolations

Phenolic compounds were isolated from 50 mg of dry leaves, which were pulverized in an MM 400 mixer mill (Retch, Germany). After the addition of 1 mL of 80% methanol (Sigma Aldrich, Czechia), samples were sonicated at room temperature for 10 min. Following centrifugation at 12,400 rcf for 5 min, the supernatant was transferred into another vial, and re-extraction was performed, again using 1 mL of 80% methanol. Extracts were stored at −20 °C until analysis. Each extract was isolated in triplicate.

Additionally, to obtain essential oil, the same plant material was subjected to hydro-distillation (1.5 h) on a Clevenger apparatus. The oils were kept at 4 °C until analysis.

### 3.3. Analysis of Mentha Extracts

The total phenolic content was spectrophotometrically determined according to the method of Singleton and Rosi [71]. The data were calculated according to a standard curve of gallic acid (25–1000 μg/mL) and expressed as gallic acid equivalents (GAE) per gram of dry weight. Methanolic extracts (10-25 μL)were diluted with 6 mL of distilled water in a 10 mL volumetric flask; then, a 500 μL of Folin–Ciocalteu reagent (diluted with water 2:1, v:v right before use) was added, and the mixture was incubated for 5 min at room temperature. Then, 1.5 mL of 20% sodium carbonate solution was added, and the samples were made up to 10 mL with distilled water. The samples were incubated in the dark for 2 h, and the absorbance was spectrophotometrically measured at 765 nm (Spectrophotometer UV/VIS S-20, Boeco, Germany) against a blank. All measurements were performed in triplicate.

Flavonoid content was estimated via the spectrophotometric method of Nagy and Grancai [72]. The data were calculated according to a standard curve of quercetin (1–50 μg/mL) and expressed as quercetin equivalents (QE) per gram of dry weight. Methanolic extracts (10–50 μL) were mixed with 500 μL of 2% aluminum chloride and 200 μL of a 1 M potassium acetate solution. After incubation for 30 min at room temperature, the absorbance was spectrophotometrically measured at 765 nm (Spectrophotometer UV/VIS S-20, Boeco, Germany) against a blank. All measurements were performed in triplicate.

UHPLC–MS/MS analyses were carried out using an Ultra Performance LCMS 8050 system (Shimadzu, Japan) with a triple quadrupole mass spectrometer equipped with electrospray ionization (ESI) source operating in the negative mode. Lab Solutions software version 5.2 (Shimadzu Corporation, Japan) was used for instrument control, data acquisition, and processing. The sample solutions were injected into a reversed-phase column (Acquity BEH C18, 1.7 μm, 3.0 × 150 mm, Waters, Milford, MA, USA) with an appropriate pre-column. The separation and identification of phenolic compounds were performed according to the conditions described before [70]. Briefly, the mobile phase consisted of a mixture of aqueous solutions of 0.1% formic acid in water (solvent A) and 0.1% formic acid in methanol (solvent B) at a flow rate of 0.4 mL/min. The column temperature was maintained at 40 °C. The linear gradient consisted of 5% B for 3 min, 5–25% B for 4 min, 25–30% B for 6 min, 30–35% B for 4 min, 35–60% B for 6 min, 60–100% for 4 min, isocratic for 1.5 min, back to 5% B within 0.1 min, and equilibration for 3.4 min. The back pressure ranged from 45 to 50 MPa during the chromatographic run. The effluent was introduced into an electrospray source (interface temperature of 300 °C, heat block temperature of 400 °C, and capillary voltage of 3.0 kV). Argon was used as the collision gas, and nitrogen was used as the nebulizing gas. To achieve a high specificity in addition to the high sensitivity, the analysis was performed in the multiple reaction monitoring (MRM) mode.

Standard solutions of the 33 target compounds were first prepared in methanol at 1 mM concentrations, and solutions were gradually diluted in the mobile phase to the working concentrations that ranged from 0.01 to 50 μM. Each solution contained 16 phenolic acids, (caffeic, chlorogenic, trans-cinnamic, 2,3-dihydroxybenzoic, ferulic, gallic, 3-hydroxybenzoic, 4-hydroxybenzoic, 5-hydroxyferulic, *p*-coumaric, protocatechuic, rosmarinic, salicylic, sinapinic, syringic, and vanillic acid), 2 phenolic acid derivatives (p-methyl coumarate and salicylic acid 2-*O*-β-D-glucoside), and 15 flavonoids (apigenin, chrysin, galangin, kaempferol, myricetin, naringenin, pinocembrin, catechin, morin, hesperidin, rutin, quercitrin, naringin, luteolin, and quercetin). Each methanolic extract (50 μL) was evaporated to dryness and redissolved into 200 μL of the mobile phase, sonicated for 5 min, and centrifuged for 5 min at 12,400 rcf before injection. All standards and reagents were of the highest available purity and purchased from Sigma Aldrich Company (Czechia), and the measurements were performed in triplicate.

The composition of essential oil constituents was analyzed via GC–MS using an Agilent 7890A gas chromatograph fitted with a fused silica HP-5MS UI (5% phenyl methyl siloxane) capillary column (30 m × 0.25 mm, 0.25 μm film thickness) coupled to an Agilent 5975C mass selective detector. All details about GC–MS conditions were described before [70]. The identification of the constituents was accomplished by visual interpretation, a comparison of their retention indices and mass spectra with literature data [73], and a computer library search (Mass Finder 4 Computer Software). Briefly, the column temperature was set from 60 to 240 °C at a heating rate of 3 °C/min, and helium was used as carrier gas (1.1 mL/min). The other operating conditions were as follows: inlet pressure of 9.43 psi, injector temperature of 250 °C, detector temperature of 280 °C, split ratio for essential oils of 1:50, and injection volume of 1 μL. A mass selective detector was operated in the electron impact (EI) mode at an ionization energy of 70 eV, a scan range of 39–550 amu, and a scan time of 1.60 s. The linear retention indices (RI) for all compounds were determined by the Kovats method by the injection of homologous series of C8–C26 *n*-alkanes in *n*-hexane solution.

A headspace sample of each *Mentha* species was directly collected via the static headspace method using a GC PAL autosampler 80 (Agilent Technologies, Santa Clara, CA, USA) equipped with a thermostated agitator and headspace syringe (maximum volume 2.5 mL). Dry and crushed plant material (5 g) was placed into a 20 mL headspace vial, which was shaken in an agitator at 40 °C. After incubation for 15 min, 1 mL of headspace was directly injected into the GC–MS instrument and analyzed as described before [70].

All standards, solvents, and reagents were of the highest available purity and purchased from Sigma Aldrich Company (Nusle, Czechia), and all of the measurements were performed in triplicate.

### 3.4. Assessment of Biological Activities

The radical scavenging ability of the isolated methanol extracts and essential oils was determined via the method of Brand-Williams et al. [74], with slight modifications [70]. The results are presented as Trolox equivalents (TE) per dry weight of the methanolic extract or mL of the essential oil. Briefly, 50 μL of essential oil were dissolved in 950 μL of dimethyl sulfoxide, and the methanolic extracts were assayed without any further preparation. A portion of sample solution (50 μL) was mixed with 1 mL of 5.25 × 10^−5^ M DPPH· in absolute ethanol. Absolute ethanol was used to zero the spectrophotometer; a DPPH· solution was used as the blank sample, and Trolox was used as a positive probe (calibration range of 0.01–0.25 mg/mL).

The tyrosinase inhibitory activity of methanolic extracts and essential oils was measured according to the protocol of Saghaie et al. [75] with some modifications. Details were described before [70]. Results are presented as kojic acid equivalents (KAE) per dry weight of the methanolic extract or mL of the essential oil. Samples were prepared as described above. Briefly, the reaction mixture contained 600 μL of a phosphate buffer (20 mM and pH 6.8), 200 μL of a tyrosinase solution (46.5 U/mL), and 10 μL of a sample solution. After incubation at 25 °C for 10 min, a 100 µL L-DOPA (L-3,4-dihydroxyphenylalanine) solution (0.85 M) was added to the mixture and incubated at 25 °C for another 20 min, and absorbance at 492 nm was read. The blank sample consisted of 800 μL of a phosphate buffer and 200 μL of a tyrosinase solution. The measurements were performed in triplicate.

All standards and reagents were of the highest available purity and purchased from Sigma Aldrich Company (Czechia).

### 3.5. Statistical Analysis

The metabolomic profile was generated and analyzed by multivariate statistical analysis. A heatmap was constructed using RStudio (Version 1.1.463–© 2021–2018 RStudio, Inc., Boston, MA, USA) using the *gplots*, *cluster*, *tidyverse*, *factoextra*, and *heatmap.plus* packages. Correlation matrices and significance were also performed in RStudio using the *factoextra*, *FactoMineR*, and *corrplot* packages.

## 4. Conclusions

The present study evaluated the phenolic profile, antioxidant properties, and enzyme inhibitory properties of thirteen *Mentha* species of European origin, all grown under the same environmental conditions. Overall, the results demonstrated that the majority of investigated plants represent promising sources of food rich in natural antioxidants, as well as sources of active ingredient agents for the pharmaceutic and cosmetic industries. Data showed that not only *M. spicata*, *M.* x *piperita*, and *M. arvensis*, which are already implemented in food and pharmaceutical industries, but also other *Mentha* species, such as *M. longifolia*, *M. microphylla*, and *M.* x *villosa* that are cultivated for ornamental purposes, have beneficial properties for humans. Citrus-scented *Mentha* x *villosa* might a good candidate, especially due to its easy cultivation.

## Figures and Tables

**Table 1:** Literature overview on phenolic compounds in selected *Mentha sp*. of European origin.

Species	Origin	TPC ^1^ (mg/g)	TFC ^2^ (mg/g)	Major Phenolics	DPPH ^3^ (IC_50_ μg/mL)	Ref.
*Mentha aquatica*	Finland	152.5 ^4^		rosmarinic acid, luteolin glucoside	275	[12]
	Greece	172–185 ^4^			29 ± 1.5	[25]
	Italy	337 ± 2.15 ^5^	15.75 ± 0.25 ^6^			[26]
	Portugal			rosmarinic acid, luteolin glucoside	8.1 ± 1.3	[27]
	Romania	212.9 ± 0.00 ^4^	0.27 ± 0.00 ^6^	rosmarinic acid, caffeic acid		[28]
*Mentha arvensis*	Finland			rosmarinic acid		[29]
*Mentha cervina*	Italy			rosmarinic acid	0.21 ± 0.01	[30]
	Portugal	0.15 ± 0.00 ^4,7^		chlorogenic acid, caffeic acid		[31]
*Mentha longifolia*	Croatia			rosmarinic acid, chlorogenic acid	8.43 ± 0.28	[32]
	Greece	115–216 ^4^	36 ± 0.6 ^7^			[25]
	Hungary	19.35–47.52 ^3^		rosmarinic acid, rutin, apigenin		[33]
	Poland			rosmarinic acid		[34]
	Romania	48 ^4^	18 ^7^			[35]
	Slovakia			rutin, kaempferol		[36]
				lithospermic acid, rosmarinic acid	25.39	[37]
*Mentha microphylla*	Greece	217–233 ^3^			29 ± 1.2	[25]
*Mentha pulegium*	Greece	138–188 ^4^			28 ± 1.0	[25]
		82.9 ± 0.1 ^4^	13.2 ± 1.8 ^8^	caffeic acid, thymol, carvacrol	179.5 ± 4.9 ^9^	[38]
	Portugal			rosmarinic acid	23	[39]
		81.2 ± 7.6			8.9 ± 0.2	[40]
	Serbia	25 4	0.05%		19.27	[41]
*Mentha x piperita*	Bulgaria	45.22 ± 0.10 ^4^				[42]
		35.1 ± 1.2 ^4^	12.5 ± 0.4 ^6^		250 9	[43]
	Croatia			rosmarinic acid	8.88 ± 0.13	[32]
	Czechia	63.0 ^4^			147.5 ^9^	[44]
	Finland	1142.4 ± 10.7 ^4^	709.3 ± 24.0 ^6^	eriocitrin, rosmarinic acid		[45]
	Germany			eriodyctiol, luteolin		[46]
	Poland			eriodyctiol, luteolin, rosmarinic acid		[34,47]
		5.58–36.04 ^4^				[48]
	Portugal			eriodyctiol, luteolin, rosmarinic acid		[49]
	Serbia	50.05 ± 0.31 ^4^	25.95 ± 1.16 ^6^		98.43 ± 2.39 ^10^	[50]
	Slovakia			rutin, gallic acid, epicatechin		[36]
				rosmarinic acid, lithospermic acid	28.45	[37]
	Spain			eriodyctiol, hesperidin, rosmarinic acid		[51]
*Mentha spicata*	Cyprus	18.91 ± 0.20 ^4^			90 ^8^	[52]
	Finland	214 ^4^		eriocitrin, luteolin, rosmarinic acid	210	[12]
	Italy		9.6 ± 0.4 ^6^	catechin, caffeic acid, rutin		[53]
	Portugal	81.2 ± 7.6 ^4^			65.2 ± 0.1	[40]
				rosmarinic acid	546 ± 17	[54]
		87.06 ± 4.56 ^4^		rosmarinic acid, hesperetin	8.93 ± 0.27	[55]
*Mentha suaveolens*	Slovakia	2.25 ± 0.297 ^4^	3.9 ± 0.001 ^6^	cinnamic acid, chlorogenic acid		[56]
*Mentha x villosa*	Slovakia			rosmarinic acid, luteolin glucoside	7.1 ± 0.09	[57]
				rosmarinic acid, lithospermic acid	39.48	[37]

^1^ Total phenolic content; ^2^ total flavonoid content; ^3^ DPPH antioxidant activity; ^4^ gallic acid equivalents; ^5^ chlorogenic acid equivalents; ^6^ quercetin equivalents; ^7^ mg gallic acid equivalents/L; ^8^ rutin equivalents; ^9^ mg Trolox equivalents/g; ^10^ mg ascorbic acid equivalents/g.

**Table 2:** Antioxidant and tyrosinase inhibitory activities of selected *Mentha* sp.

Species	Antioxidant Activity (DPPH)		Tyrosinase Inhibitory Activity (TIA)	
	Methanolic extract (mg TE ^1^/g)	Essential Oil (mg TE ^1^/mL)	Methanolic Extract (μg KAE ^2^/g)	Essential Oil (mg KAE ^2^/mL)
*M. aquatica*	67.70 ± 7.65	43.86 ± 0.33	38.45 ± 1.75	1.42 ± 0.19
*M. arvensis*	54.65 ± 5.30	7.83 ± 0.21	41.60 ± 2.61	54.36 ± 12.09
*M. cervina*	22.79 ± 1.85	20.96 ± 0.19	91.77 ± 6.11	56.61 ± 7.21
*M. longifolia*	91.01 ± 8.95	22.10 ± 0.56	50.36 ± 6.70	1.73 ± 0.15
*M. microphylla*	57.71 ± 6.42	7.28 ± 0.10	40.97 ± 3.45	164.06 ± 22.28
*M. x piperita*	85.90 ± 8.53	2.85 ± 0.38	102.82 ± 15.16	42.01 ± 6.55
*M. x piperita* Bergamot	90.60 ± 1.48	3.15 ± 0.68	36.11 ± 6.47	636.97 ± 32.58
*M. x piperita* var. *citrata*	84.34 ± 6.54	2.71 ± 0.34	43.51 ± 0.89	120.58 ± 19.40
*M. x piperita* Perpeta	52.63 ± 5.48	7.09 ± 0.89	75.74 ± 1.06	26.87 ± 7.54
*M. pulegium*	68.24 ± 3.34	40.01 ± 0.86	123.89 ± 5.64	403.19 ± 55.93
*M. spicata*	88.96 ± 10.38	14.70 ± 0.56	56.29 ± 7.21	1.33 ± 0.21
*M. suaveolens*	91.65 ± 2.92	7.11 ± 0.15	43.01 ± 1.10	40.31 ± 3.41
*M. x villosa*	106.04 ± 3.26	8.01 ± 0.74	49.83 ± 6.12	75.63 ± 9.95

^1^ Trolox equivalents; ^2^ kojic acid equivalents.

**Table 3:** Morphological characteristics of studied *Mentha* species.

Species	ECN	Collection code	Origin	Plant Height	Leaves					Corolla Color
					Petiole/Sessile	Shape	Margin	Surface	Hairiness	
*Mentha aquatica*	-	4348	Switzerland	30–40	shortly petiolate	ovate	serrate	smooth	glabrous	lilac
*Mentha arvensis*	09A6400048	3422	Slovakia	15–20	shortly petiolate	lanceolate-ovate	serrate	smooth	hairy	lilac
*Mentha cervina*	09A6400017	2112	Germany	20–25	sessile	linear-oblanceolate	obscurely toothed	smooth	glabrous	white
*Mentha longifolia*	-	4641	Slovakia	60–70	sessile	oblong-elliptical	serrate	smooth	hairy	lilac
*Mentha microphylla*	09A6400054	4344	Switzerland	50–60	sessile	lanceolate	serrate	slightly rugose	tomentose	lilac
*Mentha x piperita*	-	4371	Czechia	30–35	long petiolate	lanceolate	serrate	smooth	glabrous	lilac-pink
*Mentha x piperita* Bergamot	-	4338	Switzerland	15–20	long petiolate	subcordate	serrate	smooth	glabrous	lilac
*Mentha x piperita* var. *citrata*	-	4354	Switzerland	15–20	long petiolate	subcordate	serrate	smooth	glabrous	lilac
*Mentha x piperita* Perpeta	09A6400045	3433	Czechia	35–40	long petiolate	lanceolate	serrate	smooth	glabrous	lilac-pink
*Mentha pulegium*	-	3522	Slovenia	20–29	shortly petiolate	narrowly-elliptical	entire	smooth	subglabrous	lilac
*Mentha spicata*	09A6400015	2081	Germany	50–60	petiolate	lanceolate-ovate	serrate	smooth	glabrous	lilac
*Mentha suaveolens*	09A6400052	4340	Switzerland	50–60	sessile	ovate-oblong	serrate	rugose	hairy	pinkish
*Mentha x villosa*	-	4347	Switzerland	80–100	shortly petiolate	broadly ovate	irregular serrate	slightly rugose	hairy	pink

## Supplementary Materials

The following are available online at https://www.mdpi.com/2223-7747/10/3/550/s1. Table S1: Total phenolic and flavonoid content of methanolic extracts of selected *Mentha* sp.; Table S2: Phenolic composition (g/g) of selected *Mentha* species; Table S3: Chemical composition of the essential oils and headspace samples of selected *Mentha* species.
