# Supplementary material for: Phenolic Compounds and Biological Activity of Selected Mentha Species
Source: Plants (Basel). 2021 Mar 15;10(3):550. doi: 10.3390/plants10030550 (PMC8000339; doi:10.3390/plants10030550)
Supplement: Supplementary file 1 [file plants-10-00550-s001.zip › Supplementary files/Table S1.pdf]

Table S1. Total phenolic and flavonoid content of methanolic extracts of selected *Mentha* sp.

Species	Total phenolic content (mg GAE[1]/g)	Total flavonoid content (mg QE[2]/g)
M. aquatica	43.32 ± 3.19	14.42 ± 1.34
M. arvensis	35.96 ± 1.01	6.71 ± 0.76
M. cervina	14.81 ± 1.09	3.65 ± 0.37
M. longifolia	37.38 ± 2.52	16.83 ± 1.45
M. microphylla	40.26 ± 4.14	5.47 ± 0.76
M. x piperita	49.89 ± 5.43	14.12 ± 1.55
M. x piperita Bergamot	51.97 ± 3.26	14.42 ± 0.97
M. x piperita citrata	35.96 ± 2.06	14.48 ± 0.75
M. x piperita Perpeta	29.07 ± 4.35	5.90 ± 0.44
M. pulegium	46.39 ± 3.05	5.16 ± 0.33
M. spicata	47.92 ± 5.02	6.11 ± 0.87
M. suaveolens	58.93 ± 8.39	8.28 ± 0.62
M. villosa	52.61 ± 6.38	15.77 ± 0.84

[1]Gallic acid equivalents; [2]Quercetin equivalents.
