# Supplementary material for: Phenolic Compounds and Biological Activity of Selected Mentha Species
Source: Plants (Basel). 2021 Mar 15;10(3):550. doi: 10.3390/plants10030550 (PMC8000339; doi:10.3390/plants10030550)
Supplement: Supplementary file 1 [file plants-10-00550-s001.zip › Supplementary files/Table S2.pdf]

Table S2. Phenolic composition (µg/g) of selected *Mentha* species.

	Compound	*M. aquatica*	*M. arvensis*	*M. cervina*	*M. longifolia*	*M. microphylla*	*M. x piperita*	*M. x piperita Bergamot*	*M. x piperita* var. *citrata*	*M. x piperita* Perpeta	*M. pulegium*	*M. spicata*	*M. suaveolens*	*M. x villosa*
Hydroxybenzoic acids	GA[1]	0.39	0.22	0.09	0.57	0.16	0.63	0.25	1.10	0.30	0.25	0.23	0.24	0.20
		±0.01	±0.03	±0.01	±0.01	±0.01	±0.01	±0.01	±0.01	±0.03	±0.02	±0.01	±0.01	±0.02
	4HBA[2]	0.42	2.73	0.71	0.411	0.83	0.51	0.80	0.68	1.98	1.75	0.50	0.76	1.70
		±0.01	±0.10	±0.03	±0.02	±0.01	±0.02	±0.03	±0.02	±0.07	±0.06	±0.04	±0.04	±0.06
	3HBA[3]	1.52	3.65	4.44	2.82	2.81	2.70	3.4	1.75	2.47	2.70	0.72	1.00	2.32
		±1.06	±0.65	±0.32	±0.45	±0.59	±1.01	8±1.01	±0.84	±0.69	±1.64	±0.10	±0.19	±0.12
	23DHBA[4]	5.21	10.55	1.31	3.12	5.14	3.96	8.60	2.41	9.41	9.15	4.21	5.25	8.49
		±1.31	±0.11	±0.04	±0.46	±1.68	±0.04	±1.49	±0.28	±2.90	±0.20	±0.72	±0.17	±0.28
	VA[5]	2.39	2.47	5.95	2.79	2.71		2.57	2.45	2.18	3.04	8.72	8.71	3.93
		±0.72	±0.70	±1.19	±0.36	±0.56	n.d.[22]	±0.89	±0.56	±0.50	±0.26	±3.83	±0.46	±0.93
	SaA[6]	1.74	7.05	1.80	2.51	1.90	1.12	1.97	0.76	6.57	7.36	0.94	4.38	6.46
		±0.09	±0.11	±0.06	±0.05	±0.03	±0.08	±0.04	±0.04	±0.03	±0.20	±0.04	±0.10	±0.09
	SaAG[7]	26.23	149.79	14.01	21.88	18.96	31.15	50.61	15.87	117.46	110.61	3.27	44.58	108.55
		±1.12	±8.14	±0.46	±0.87	±0.82	±0.45	±2.08	±0.35	±7.66	±4.88	±0.16	±1.67	±0.90
Hydroxycinnamic acids	CA[8]	9.48	20.50	9.85	17.27	26.87	16.70	20.80	26.82	12.76	33.48	14.11	28.23	26.55
		±0.16	±0.31	±0.23	±0.38	±0.39	±0.49	±0.11	±0.41	±0.08	±0.41	±0.21	±0.56	±0.56
	ChA[9]	44.84	36.04	12.82	30.06	28.18	21.38	18.13	16.39	23.02	32.47	38.78	20.41	24.03
		±0.47	±1.25	±0.69	±0.91	±0.90	±0.94	±0.09	±0.15	±0.90	±0.83	±1.30	±0.32	±0.98
	pCA[10]	2.91	2.06	1.4	2.53	4.12	1.80	2.50	2.34	1.89	3.98	5.52	6.15	5.04
		±0.05	±0.06	7±0.04	±0.06	±0.05	±0.04	±0.04	±0.04	±0.01	±0.04	±0.19	±0.13	±0.12
	FA[11]	3.54	3.70	1.50	4.81	6.53	5.33	9.50	9.63	7.60	6.94	4.57	9.14	6.19
		±0.12	±0.06	±0.04	±0.09	±0.42	±0.12	±0.32	±0.12	±0.06	±0.24	±0.21	±0.08	±0.36
	RA[12]	1666.24	1960.61	1363.38	1872.68	2111.51	1816.12	2144.24	1622.97	1787.50	2442.37	1870.07	2557.08	2298.86
		±42.93	±31.19	±83.23	±43.35	±70.24	±8.18	±43.97	±9.14	±17.49	±81.23	±5.65	±64.21	±28.49
	tCA[13]	4.83	1.84	0.71	0.90	1.14	2.87	3.16	1.41	1.44	0.42	0.21	0.70	0.68
		±0.18	±0.07	±0.36	±0.70	±1.22	±0.28	±1.26	±0.19	±0.59	±0.46	±0.02	±0.67	±0.56
	pMCA[14]	0.44	0.54	0.12	0.68	0.51	0.51	0.27	0.62	0.34	0.49	0.38	1.17	2.20

		±0.02	±0.02	±0.00	±0.01	±0.01	±0.02	±0.02	±0.01	±0.01	±0.02	±0.01	±0.03	±0.03
Flavonoids	HES[15]	84.57 ±0.43	121.95 ±2.35	1.68 ±0.03	12.98 ±0.16	10.71 ±0.17	109.39 ±2.01	61.80 ±0.51	19.45 ±0.06	96.70 ±1.72	7.74 ±0.01	0.73 ±0.02	18.88 ±0.25	3.87 ±0.06
	RUT[16]	6.95 ±1.72	5.79 ±0.57	35.15 ±0.34	7.67 ±1.50	5.05 ±0.32	11.73 ±0.52	11.91 ±2.66	4.89 ±0.29	7.09 ±0.30	14.22 ±1.02	26.31 ±1.09	25.75 ±1.57	10.49 ±4.17
	QUE[17]	0.09 ±0.03	0.10 ±0.01	0.05 ±0.00	0.09 ±0.02	n.d.	n.d.	0.10 ±0.01	0.05 ±0.00	0.06 ±0.02	0.06 ±0.01	0.05 ±0.00	0.04 ±0.00	0.10 ±0.00
	NAR[18]	2.37 ±0.20	6.86 ±0.58	2.52 ±0.14	0.11 ±0.00	2.72 ±0.24	2.36 ±0.15	0.57 ±0.02	1.07 ±0.01	2.69 ±0.07	1.39 ±0.06	1.33 ±0.07	1.8 1±0.17	1.50 ±0.05
	LUT[19]	12.97 ±0.69	6.17 ±0.46	17.23 ±1.40	28.33 ±1.28	1.84 ±0.11	8.60 ±0.32	31.03 ±1.16	18.95 ±0.38	3.19 ±0.05	6.75 ±0.20	2.09 ±0.11	19.62 ±0.17	22.21 ±1.00
	KAE[20]	16.85 ±1.04	6.55 ±0.61	21.65 ±1.50	33.68 ±0.81	1.30 ±0.17	10.43 ±0.33	41.47 ±2.35	21.63 ±0.51	3.05 ±0.07	6.44 ±0.26	1.81 ±0.24	22.17 ±0.63	26.50 ±1.90
	API[21]	1.87 ±0.01	3.98 ±0.07	5.07 ±0.13	1.82 ±0.07	2.28 ±0.00	1.27 ±0.02	8.22 ±0.19	2.09 ±0.04	1.02 ±0.01	2.03 ±0.01	0.33 ±0.02	3.53 ±0.03	2.15 ±0.04

[1]Gallic acid; [2]4-Hydroxybenzoic acid; [3]3-Hydroxybenzoic acid; [4]2,3-Dihydroxybenzoic acid; [5]Vanillic acid; [6]Salicylic acid; [7]Salicylic acid 2-*O*-β-glucoside; [8]Caffeic acid; [9]Chlorogenic acid; [10]*p*-Coumaric acid; [11]Ferulic acid; [12]Rosmarinic acid; [13]*trans*-Cinnamic acid; [14]*p*-Methyl coumarate; [15]Hesperidin; [16]Rutin; [17]Quercetin; [18]Naringenin; [19]Luteolin; [20]Kaempferol; [21]Apigenin; [22]not detected.
