# Supplementary material for: Phenolic Compounds and Biological Activity of Selected Mentha Species
Source: Plants (Basel). 2021 Mar 15;10(3):550. doi: 10.3390/plants10030550 (PMC8000339; doi:10.3390/plants10030550)
Supplement: Supplementary file 1 [file plants-10-00550-s001.zip › Supplementary files/Table S3.pdf]

Table S3. Chemical composition of the essential oils and headspace samples of selected *Mentha* species.

		M. aquatica		***M. arvensis***		***M. cervina***		***M. longifolia***		***M. microphylla***		***M. x piperita***		***M. x piperita bergamot***		***M. x piperita citrata***		***M. x piperita perpeta***		***M. pulegium***		***M. spicata***		***M. suaveolens***		***M. villosa***	
**RI**	**Compound**	**HS**	**EO**	**HS**	**EO**	**HS**	**EO**	**HS**	**EO**	**HS**	**EO**	**HS**	**EO**	**HS**	**EO**	**HS**	**EO**	**HS**	**EO**	**HS**	**EO**	**HS**	**EO**	**HS**	**EO**	**HS**	**EO**
925	α-Thujene	0.3						11.7	3.0																		
932	α-Pinene	9.1	2.0	5.9	1.2	11.6	0.9	3.4	1.1	4.4	1.0	7.5	0.8			2.3	0.2	4.4	0.7	6.3	0.2	5.5	1.2	14.9	1.1	3.5	0.5
947	Camphene	1.5	0.3																								
972	Sabinene	4.6	1.5	3.3	1.2	2.9	0.4	4.6	2.0	2.9	0.8	4.3	0.4	0.5		3.1	0.4	1.9	0.5			3.5	1.2	4.7	0.5	2.2	0.5
976	β-Pinene	8.2	3.1	24.3	8.1	7.2	1.3	3.1	2.1	7.9	1.9	6.3	1.0	1.0	0.3	3.8	0.5	0.1	1.1	2.7	0.3	6.1	2.2	12.0	2.8	2.8	0.9
990	Myrcene	1.8	0.6	2.2	0.4	3.4	0.6	6.2	2.5	4.9	1.2	1.9	0.2	2.7	1.2	3.5	1.4	2.1	0.4			44.2	10.3	8.9	1.3	3.3	0.8
995	3-Octanol									0.5	0.6								0.2	0.2	1.8		0.3			0.4	0.4
1017	α-Terpinene							4.1	3.2			0.2	0.1														
1024	*p*-Cymene	0.5						26.0	9.2			0.3				0.2											
1027	Limonene	6.3		52.7	14.4	36.8	6.4			43.0	9.7	8.1	1.4	2.3	0.4	1.5	0.7	8.4	1.5	4.3	0.2	6.0	1.6	28.4	4.8	60.2	17.7
1030	1,8-Cineole	35.3	22.9	2.1	1.4			25.1	25.0	15.2	6.8	26.7	5.7	5.0	1.3	23.3	4.9	9.3	4.2			19.5	12.0			8.2	2.9
1036	(*Z*)-β-Ocimene			1.1	0.3			1.6	1.5	2.0	0.9	0.5		0.5		1.4	0.7	0.6	0.4			4.2	2.1	7.5	1.9	2.7	1.0
1046	(*E*)-β-Ocimene			0.3	0.4										0.9		0.7										
1057	γ–Terpinene	0.2	0.2					14.2	12.1				0.3				0.2										
1065	*cis*-Sabinene hydrate	0.1			0.4				0.4			2.2	1.4			0.3			0.2								
1087	Terpinolene																0.3										
1087	Fenchone						0.4								0.3								0.4				0.8
1100	*trans*-Sabinene hydrate		0.5		0.5						0.8												0.2				
1103	Methyl benzoate															0.2											
1104	Linalool	0.2										2.5	2.9	38.6	45.4	23.8	33.5					1.5		1.7			
1112	1-Octen-3-yl acetate														0.4	1.0	0.5							2.8	2.7		
1123	3-Octanol acetate								0.7							2.0							0.3				

1153	Menthone	6.6	10.8			0.1	12.1					8.1	7.0	0.6				38.8	37.4	22.0	11.7						
1163	Isoborneol											0.5				0.2											
1163	Menthofuran	24.4	51.5		0.5							10.1	16.5					25.6	20.8		11.0						
1165	δ-Terpineol								0.7										5.9				0.4				
1170	*iso*-Menthone					19.5				0.7										15.2							
1176	Menthol												46.4						16.0		1.2						
1176	Terpinen-4-ol				0.5				0.2			15.1	0.6			0.7	0.3	5.7	0.3								
1184	*iso*-Menthol											0.2							0.3		2.4						
1189	*neoiso*-Menthol												0.3														
1190	α-Terpineol	0.2	1.3		0.4				2.0		1.0		0.6		5.8		4.8						0.4				
1195	Thymol								18.6																		
1197	*cis*-Dihydro carvone			1.2	5.8					5.3	16.1															2.9	10.6
1227	Nerol								0.4						1.3		0.6										
1237	Pulegone	0.3	1.3		2.7	14.7	39.3						0.5	1.1				2.1	4.8	49.4	69.6						
1248	Carvone			7.1	55.9					12.7	54.2														6.0	13.8	58.7
1254	Piperitone				0.8		6.4				0.6								0.8								0.6
1258	Linalool acetate											2.5	2.4	47.8	33.1	30.0	29.2					3.4		4.6			
1261	*cis*-Carvone oxide																						0.4		0.7		
1274	*neo*-Menthyl acetate												0.4						1.2		0.3						
1293	Menthyl acetate											2.0	6.2					0.5	2.1								
1302	Carvacrol								1.0																		
1343	Piperitenone				3.3	3.9	30.4																				
1350	α-Terpinyl acetate												1.2			2.6	13.7										
1353	Thymol acetate								0.6																		
1364	Neryl acetate														2.1		1.2										
1372	Piperitenone oxide								0.5													6.2	47.7	14.6	60.7		0.5
1380	Geranyl acetate										0.9				3.4		2.2										
1391	β-Elemene																						1.5		3.0		
1417	(*E*)-Caryophyllene		0.8		1.2		0.6		4.7		1.3		1.2		1.0		1.1		0.6				3.1		1.1		1.3
1444	*cis*-Muurola-3,5-diene																						0.6				

1450	α-Humulene								0.3																		
1457	(*E*)-β-Farnesene																						0.7		1.3		
1460	*cis*-Cadina-1(6),4-diene																						0.7				
1478	γ-Muurolene		0.5						3.1		1.0		1.4		0.5		1.0		0.5		0.5		5.0		8.3		
1494	Bicyclogermacrene																						1.5				
1521	δ-Cadinene		0.3																				0.4				
1547	Elemol																1.3										
1588	Viridiflorol												0.4		0.9										3.0		
	Yield		1.3		0.7		1.3		1.0		1.5		1.5		1.2		3.3		1.0		1.0		0.7		0.5		1.9
	Total identified	99.7	97.6	100.0	99.4	100.0	98.5	100.0	95.1	99.5	99.3	99.4	99.5	100.0	98.2	99.7	99.2	99.5	99.8	100.0	99.4	100.0	94.2	100.0	99.3	100.0	98.6
